# Ursolic acid, a dietary phytochemical, decreases KRAS signaling and modulates cell death pathways in resistant CRC cells

**DOI:** 10.1186/1753-6561-6-S3-P38

**Published:** 2012-06-01

**Authors:** Cristina Xavier, Cristóvão Lima, Dalila Pedro, Cristina Pereira-Wilson

**Affiliations:** 1 CBMA/Department of Biology, University of Minho, 4710-057 Braga, Portugal; 2 CITAB/Department of Biology, University of Minho, 4710-057 Braga, Portugal

## 

KRAS mutations are frequent in colorectal cancer (CRC) and have the potential to activate proliferation and inhibit cell death through effects on MAPK/ERK and PI3K/Akt signaling pathways. Because diet is one of the most important determinants of CRC incidence and progression, we studied the effects of the dietary triterpenoid ursolic acid (UA) on proliferation and cell death induction in human CRC derived KRAS mutated cell lines.

Our results show that UA decreases cell proliferation and induces cell death while decreasing signaling through KRAS as indicated by a decrease in ERK and Akt phosphorylation (western blot). UA also induced cell death.

TP53 mutated cells are known to be resistant to the chemotherapeutic drug 5-FU. Caspase independent apoptosis (Tunel assay), was increased 6 fold by co-incubation of UA with 5-FU. However, apoptosis was only a small percentage of the total cell death induced by UA. In order to explain these observations, we looked into effects on autophagy. Autophagy is emerging as a promising therapeutic target for drug resistant tumors. UA modulated autophagy by inducing the accumulation of LC3 II and p62 levels an effect dependent on JNK activation.

In conclusion, this study shows UA’s anticancer potential as a modulator of KRAS signaling and cell death mechanisms increasing sensitivity to the chemotherapeutic drug 5-FU.

